# Inhibition of Phenolics Uptake by Ligninolytic Fungal Cells and Its Potential as a Tool for the Production of Lignin-Derived Aromatic Building Blocks

**DOI:** 10.3390/jof6040362

**Published:** 2020-12-12

**Authors:** Mathilde Leriche-Grandchamp, Amandine Flourat, Hangchen Shen, Flavien Picard, Heloïse Giordana, Florent Allais, Antoine Fayeulle

**Affiliations:** 1 AgroParisTech, CEBB, URD Agro-Biotechnologies Industrielles (ABI), 51110 Pomacle, France; 2 TIMR (Integrated Transformations of Renewable Matter), ESCOM, Centre de Recherche Royallieu, Université de Technologie de Compiègne, CS 60 319, 60203 Compiègne, France

**Keywords:** biomass valorization, carbonyl cyanide *m*-chlorophenyl hydrazine, lignin, monoaromatics, monophenols, *Phanerochaete chrysosporium*

## Abstract

Lignin is the principal natural source of phenolics but its structural complexity and variability make it difficult to valorize through chemical depolymerization approaches. White rots are one of the rare groups of organisms that are able to degrade lignin in ecosystems. This biodegradation starts through extracellular enzymes producing oxidizing agents to depolymerize lignin and continue with the uptake of the generated oligomers by fungal cells for further degradation. *Phanerochaete chrysosporium* is one of the most studied species for the elucidation of these biodegradation mechanisms. Although the extracellular depolymerization step appears interesting for phenolics production from lignin, the uptake and intracellular degradation of oligomers occurring in the course of the depolymerization limits its potential. In this study, we aimed at inhibiting the phenolics uptake mechanism through metabolic inhibitors to favor extracellular oligomers accumulation without preventing the ligninases production that is necessary for extracellular depolymerization. The use of sodium azide confirmed that an active transportation phenomenon is involved in the phenolics uptake in *P. chrysosporium*. A protocol based on carbonyl cyanide *m*-chlorophenyl hydrazone enabled reaching 85% inhibition for vanillin uptake. This protocol was shown not to inhibit, but on the contrary, to stimulate the depolymerization of both dehydrogenation polymers (DHPs) and industrial purified lignins.

## 1. Introduction

The valorization of lignin is important for making second-generation biorefineries economically viable [1]. Nonetheless, because of its high structural diversity, complexity, and stability, there is nearly no economically viable way of valorizing lignin apart from using this polymer as a combustible to generate heat and electricity [2]. Only 1% of the annually produced lignin is being valorized for its application in the preparation of chemicals and, to a limited extent, for biomaterials [3]. 

Thus, creating high-value molecules from lignin appears to be a promising field of research. In particular, phenolic monomers show miscellaneous applications in different fields: anti-oxidant [4], anti-UV [5,6], or polymers [7]. However, today, they remain mostly derived from fossil resources. Lignin is an interesting source of non-fossil-based aromatic building blocks for chemical syntheses as it is the most abundant aromatic biopolymer. However, the major limitations of chemical lignin depolymerization are the costs and efficiencies, mainly because of the diversity of inter-unit linkages between the residues of monolignols [8], the recondensation/repolymerization of generated radical fragments, and the formation of more stable C–C bonds [9] leading often to more recalcitrant species [10]. Furthermore, a soft and controlled process of depolymerization of lignin is required to keep the aromatic structure intact. Diverse strategies have been developed for lignin depolymerization, including thermochemical treatments, homogeneous and heterogeneous catalysis, and biological depolymerization [3]. Among them, the latest strategy appears to be less energy-demanding as atmospheric pressure and ambient temperature are adequate. 

In particular, white-rot Basidiomycetes have been extensively explored and their mechanisms of lignin depolymerization have been elucidated [11]. Ligninolytic fungi are described as being able to depolymerize lignin through extracellular enzymes called ligninases, which encompass laccases, versatile, lignin- and manganese-peroxidases, dye-decolorizing peroxidases, and unspecific peroxygenases [12], as well as other oxidases/dehydrogenases enzymes, such as aryl alcohol oxidase (AAO) and glyoxal oxidase, which produce hydrogen peroxide, and fungal aryl-alcohol dehydrogenases (AAD) and quinone reductases (QR), which reduce lignin-derived compounds [13]. Monophenols or phenolic oligomers are then taken up by cells for further intracellular biodegradation and used as carbon and energy sources. This occurs through so-called funneling pathways, which lead the different aromatic molecules to take part in a ring fission pathway and to central carbon metabolism, often via acetyl-CoA [14]. The use of these biological activities to generate high-value biosourced aromatic synthons could be of interest since fungal lignin depolymerization appears to be not limited by repolymerization phenomena. Furthermore, this monomer production from lignin could be coupled to the valorization of cellulose since biopulping is now regarded as a promising cost-effective and environmentally friendly approach [15].

Among white-rot Basidiomycetes, *Phanerochaete chrysosporium* is considered as a model strain for lignin degradation as it can produce a more complete ligninolytic enzyme complex than most other strains [16]. Several catabolic pathways for the breakdown of lignin components have been described. Many of these catabolic pathways lead to the production of vanillin, or its oxidation product vanillic acid, and then, to protocatechuic acid and catechol. Nevertheless, protocatechuate and catechol, the central intermediates of the numerous aromatic catabolic pathways, undergo ring cleavage and are further converted via the β-ketoadipate pathway to central carbon metabolism [17]. As the enzymes involved in the cleavage of the aromatic ring (catechol-1,2-dioxygenase and 4-carboxymuconolactone decarboxylase) are intracellular [18], a way to allow phenolic monomers recovery is to prevent the cleavage of the aromatic ring by preventing the transportation of phenolic monomers through the plasma membrane. The uptake of phenolic compounds by fungal cells is suspected to involve active phenomena for both monoaromatic [19] and polyaromatic [20] compounds, but the mechanism involved remains unclear.

In this study, we hypothesized that the monophenols uptake by the cells of *P. chrysosporium* after lignin depolymerization by extracellular enzymes is due to an active transport phenomenon, which can be inhibited to increase the rate of monophenols recovery without impairing the ligninases production.

## 2. Materials and Methods

### 2.1. Strains and Culture Conditions

The strain *Phanerochaete chrysosporium* BRFM 127 was subcultured every week on Petri dishes containing potato dextrose agar (Condalab, Madrid, Spain) and incubated at 22 °C under a 12 h:12 h photoperiod in such a way as to obtain sporulating cultures after two weeks. Mycelium used for inhibition experiments was produced using MYPC liquid media (Malt extract—Condalab, Madrid, Spain; Peptone from soy flour—Merck, Darmstadt, Germany; Yeast extract—VWR, Fontenay-sous-Bois, France; Casamino acid—Becton Dickinson, Rungis, France) [19] supplemented with 1 g∙L^−1^ of agar (Merck, Darmstadt, Germany) to prevent pellet formation by acting on the medium’s viscosity. These cultures were carried out in 250 mL Erlenmeyer flasks containing 50 mL of MYPC medium inoculated at an initial concentration of 10^4^ spores/mL and incubated for 3 days at 22 °C under a 12 h:12 h photoperiod and orbital shaking at 180 rpm. These spore-inoculated liquid cultures were used to inoculate other MYPC liquid cultures using 5 mL to a total volume of 50 mL and incubated in the same conditions and time in order to get homogeneous actively growing mycelia. The mycelium was then harvested through filtration and homogenized by hashing with scalpels just before its use as an inoculum for monophenols uptake or lignin degradation experiments.

### 2.2. Phenolics Uptake Inhibition Experiments

Vanillin, vanillic acid, sodium azide, and carbonyl cyanide *m*-chlorophenyl hydrazone (CCCP) were purchased from Sigma-Aldrich (Saint-Quentin-Fallavier, France). These experiments were carried out within a mineral medium at pH 7 (KCl 0.250 g∙L^−1^, NaH_2_PO_4_ 6.464 g∙L^−1^, Na_2_HPO_4_·2H_2_O 10.408 g∙L^−1^, MgSO_4_.7H_2_O 0.244 g∙L^−1^, NO_3_NH_4_ 1.000 g∙L^−1^, ZnSO_4_.7H_2_O 1.000 mg∙L^−1^, MnCl_2_.4H_2_O 0.100 mg∙L^−1^, FeSO_4_.7H_2_O 1.000 mg∙L^−1^, CuSO_4_.5H_2_O 0.500 mg∙L^−1^, CaCl_2_.2H_2_O 0.100 mg∙L^−1^, MoO_3_ 0.200 mg∙L^−1^) or pH 5.5 (KCl 0.250 g∙L^−1^, NaH_2_PO_4_ 1.544 g∙L^−1^, Na_2_HPO_4_·2H_2_O 0.008 g∙L^−1^, MgSO_4_.7H_2_O 0.244 g∙L^−1^, NO_3_NH_4_ 1.000 g∙L^−1^, ZnSO_4_.7H_2_O 1.000 mg∙L^−1^, MnCl_2_.4H_2_O 0.100 mg∙L^−1^, FeSO_4_.7H_2_O 1.000 mg∙L^−1^, CuSO_4_.5H_2_O 0.500 mg∙L^−1^, CaCl_2_.2H_2_O 0.100 mg∙L^−1^, MoO_3_ 0.200 mg∙L^−1^). When required, a carbon source composed of glucose was added as a mother solution after autoclavation at a final concentration of 20 g∙L^−1^. Mineral salts and glucose were purchased from Thermo Fisher Scientific (Illkirch-Graffenstaden, France). 

CCCP is described as being insoluble in water and thus mycelia were first preincubated with this inhibitor 16 h before the vanillin addition to allow for sufficient time for the inhibitor to reach and act on its cell targets. In 50 mL Erlenmeyer flasks, 20.5 mg of CCCP was introduced in the form of a stock solution in ethanol, and ethanol was evaporated under air flux at room temperature before the addition of 10 mL of mineral medium and autoclavation. Sodium azide is water-soluble and was thus directly added to the medium composition with a final concentration of 100 mM. The controls consisted of the same setup without the inhibitor to establish the monophenols uptake by the fungus under the considered experimental conditions and in non-inoculated media to determine the monophenols abiotic losses. A total of 300 mg FW (Fresh Weight) of actively growing mycelium were inoculated per culture and all conditions were cultured for 16 h before the addition of vanillin or vanillic acid to a final concentration of 1.56 mM. Mycelia were incubated for 3 h with vanillin and the cultures were stopped through filtration. Filtrates were harvested for non-incorporated vanillin quantification and the biomasses were dried at 100 °C in an oven to be able to calculate the relative incorporated monophenols quantities. All conditions were run in triplicates.

### 2.3. Phenolics HPLC Quantification

The quantification of non-incorporated vanillin or vanillic acid was carried out using a UHPLC (Thermo Ultimate 3000) with a Syncronis aQ C18 column (Thermo Fisher Scientific, Illkirch-Graffenstaden, France). A total of 20 µL of filtrates were injected, the flow rate was 0.5 mL∙min^−1^, the column temperature was 30 °C, and the mobile phase consisted of a gradient of acetonitrile in distilled water with formic acid 1% (0% of acetonitrile for 10 min, 10% for 5 min, 15% for 30 min, and 25% for 5 min). Monophenols were detected at 285 nm. Calibration curves were established with the commercial molecules.

### 2.4. Dehydrogenase Polymers Syntheses

Coniferyl alcohol (also known as monolignol G) was synthesized, as previously described [21]. Citric acid monohydrate and sodium hydrogenophosphate heptahydrate were purchased from VWR (Fontenay-sous-Bois, France). Laccase from *Trametes versicolor* was purchased from Sigma Aldrich (Saint-Quentin-Fallavier, France). Acetonitrile, gel permeation chromatography (GPC)-grade THF, and ethyl acetate were purchased from Fisher Scientific (Illkirch-Graffenstaden, France). A polystyrene standard kit was purchased from Agilent Technologies (Les Ulis, France). GPC was performed on an Infinity 1260 system from Agilent Technologies (Les Ulis, France) equipped with a UV detector.

To obtain dehydrogenation polymers (DHPs) with 1 to 5 monomers, coniferyl alcohol (3.4 g, 18.9 mmol) was dissolved in acetonitrile (20 mL), then diluted with a citrate phosphate buffer with pH 5.6 (190 mL) under vigorous stirring and heated at 45 °C. When this temperature was reached, a solution of laccase from *Trametes versicolor* (37.8 U, 2 U/mmol) in a citrate phosphate buffer with pH 5.6 (40 mL) was added at the reaction media through a syringe pump set at 10 mL/h. At the end of the addition, the reaction media was stirred for 3 more hours (7 h total), then quenched with ethyl acetate (70 mL). The layers were separated. The aqueous layer was extracted twice with ethyl acetate (50 mL). The organic layers were combined, washed with brine (70 mL), dried over anhydrous MgSO_4_, filtered, and concentrated to afford 3.05 g of DHPs.

### 2.5. Dehydrogenation Polymers and Purified Lignins Depolymerization Experiments

Concerning the experiments with DHP, the mycelia preparation and preincubation with CCCP were carried out in the same way as for the uptake inhibition experiments in a mineral medium with pH 5.5 and a C/N ratio of 21.34. A total of 57.5 mg of DHP was introduced per culture under the form of a mother solution in DMSO (25 g∙L^−1^) after the 16 h of preincubation. Controls without the inhibitor and abiotic controls were also produced. The cultures were incubated for 72 h at 22 °C under a 12 h:12 h photoperiod and orbital shaking at 180 rpm before the oligomers quantification. Extracellular peroxidase activities were measured in the same conditions using the enzymatic assay, as previously described [22].

Regarding the experiments with Kraft lignin and Organosolv lignin, no preincubation of the mycelium with the inhibitor was carried out since lignin had to be depolymerized before the oligomers uptake letting time to CCCP to act on fungal cells. Thus, 57.5 mg of the considered purified lignin was directly added to the medium before autoclavation. Cultures were inoculated in the same way as for the other experiments and incubated for 11 days under the same conditions before the oligomers quantification. Controls without inhibitor and abiotic controls were also carried out. Kraft lignin (CAS: 8068-05-1, Sigma-Aldrich, Saint-Quentin-Fallavier, France) was obtained according to the conventional Kraft delignification that entails treatment of wood chips with a hot mixture of water, sodium hydroxide (NaOH), and sodium sulfide (Na_2_S). Unfortunately, the supplier did not provide any further information regarding the process nor the composition of the lignin. The molecular weight of the neat lignin was found to be *M_w_* = 1.6 kDa with a polydispersity of 1.9. Organosolv lignin (CAS: 8068-03-9, Chemical Point, Oberhaching, Germany) was obtained according to the conventional Organosolv delignification that consists of contacting a lignocellulosic feedstock, such as chipped wood, with an aqueous organic solvent (e.g., acetone, methanol, ethanol, butanol, ethylene glycol, formic acid, acetic acid) at temperatures ranging from 140 to 220 °C. Unfortunately, the supplier did not provide any further information regarding the process nor the composition of the lignin. The molecular weight of the neat lignin was found to be *M_w_* = 1.7 kDa with a polydispersity of 2.1. The chemical differences in lignin structures due to these two kinds of treatment were recently studied [23].

For all depolymerization experiments, cultures were stopped through filtration; then, the filtrates and filters with biomasses were harvested, frozen at −80 °C, and lyophilized before further analyses.

### 2.6. High-Performance Sized Exclusion Chromatography (HPSEC) Analyses of Lignin Oligomers

A total of 3 mg/mL ± 0.5 mg of sample was dissolved in GPC-grade THF. A total of 20 µL of the sample was injected through a PLgel column of 5 µm, 100 Å (600 × 7.5 mm) (Agilent Technologies, Les Ulis, France), at 40 °C with a flow rate of THF of 1 mL/min. UV chromatograms were recorded at 280 nm. A calibration curve was established using polystyrene standards.

### 2.7. Data Processing

Results were expressed as mean value ± standard deviation for three replicates. The statistical analysis was performed using an unpaired Student’s *t*-test (at 99% confidence).

## 3. Results and Discussion

### 3.1. Characterization of Phenolics Cellular Uptake by Phanerochaete chrysosporium

Even though the extracellular [24] and intracellular [25] metabolic pathways involved in lignin catabolism have been widely investigated, particularly in *P. chrysosporium*, the understanding of the step of the fungal plasma membrane crossing is still poorly understood. This understanding is, however, of major interest for monoaromatics bioproduction from lignin since only an active transport phenomenon can be inhibited to improve the recovery rate. 

**Figure 1:**
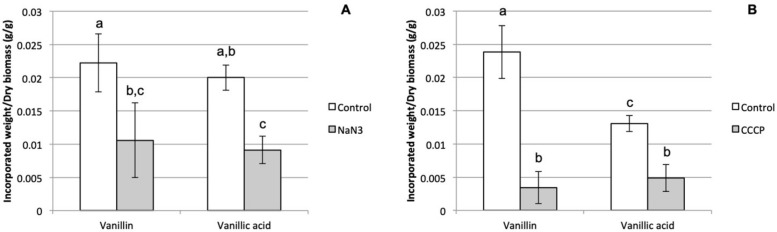
Effects of sodium azide (**A**) and carbonyl cyanide *m*-chlorophenyl hydrazone (CCCP) (**B**) on the relative incorporated quantities of vanillin and vanillic acid by *P. chrysosporium* biomasses (letters indicate groups with no significant statistical difference according to an unpaired Student *t*-test at a 99% confidence level).

The use of sodium azide, an inhibitor of ATP production, enabled a significant decrease in the uptake of two lignin derivable monophenols by *P. chrysosporium*, i.e., vanillin and vanillic acid, in comparison to the controls without the inhibitor (Figure 1A). This result is the first piece of evidence for the involvement of an active transport phenomenon in the uptake of these monophenols by this species. Furthermore, the uptake of these two phenolic compounds in the presence of glucose is in accordance with the conclusions of Barnhart-Dailey et al. [26] regarding the non-specificity of the transporters involved in the ligninolytic secondary metabolism system. Indeed, in this study, the authors quantified comparable levels of incorporation for different model lignin degradation products in cultures of *P. chrysosporium* with glucose or microcrystalline cellulose at the cellular level. 

In order to further investigate the nature of the monophenols uptake mechanism, the same type of experiment was carried out with CCCP, which is a proton-gradient uncoupler [27]. The significant decrease of both vanillin and vanillic acid uptakes by the cells of *P. chrysosporium* compared to the corresponding controls (Figure 1B) indicated that the proton motive force (PMF) must have been involved in the transport phenomenon in a direct or indirect way. Indeed, the PMF could be directly involved in vanillin and vanillic acid uptake at the plasma membrane level through a secondary active transport mechanism, as proposed by Shimizu et al. [19], who notably used CCCP on *Fomitopsis palustris* protoplasts. However, the ATP used in primary active transports is interconnected with PMF at several levels since PMF is used in mitochondria by ATP synthase for ATP synthesis and ATP is also used by proton pumps in plasma membranes to generate PMF. The fact that the inhibition with CCCP led to lower incorporated monophenols relative quantities than with sodium azide could be a sign that the phenomenon disturbed by CCCP, i.e., PMF, was more directly involved in the uptake than the ATP intracellular concentration targeted by NaN_3_. Thus, our results could be in accordance with the involvement of secondary active transport. Shary et al. [28] analyzed the proteomics of the microsomal fraction of *P. chrysosporium* under both ligninolytic (cellulose) and non-ligninolytic (glucose) conditions and detected six putative transporters. Only one of them, the protein 137220, was produced in both conditions and was upregulated under ligninolytic cultures in accordance with our observation of a phenolics uptake with glucose. This protein is assigned to the major facilitator superfamily (MFS) and has homologies with a hexose transporter of *Amanita muscaria* and a glucose sensor of *Saccharomyces cerevisiae*. Considering that the MFS superfamily corresponds to membrane transport proteins using chemiosmotic gradients to energetically drive the transport of small solutes through uniports or cotransports, and that this protein is homologous with membrane protein involved in carbon sources detection and uptake, the protein 137220 could be a good candidate to explain the monophenols uptake behavior that we observed.

### 3.2. Compatibility of Phenolics Uptake Inhibition and Ligninolytic Enzymes Production Conditions

**Figure 2:**
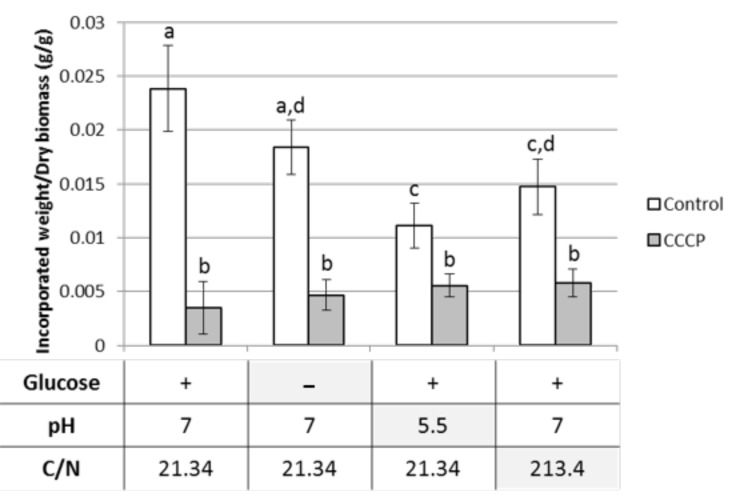
Effects of the glucose starvation, the pH acidification, and the C/N ratio increase on the CCCP inhibition of vanillin uptake by *P. chrysosporium* (letters indicate groups with no significant statistical difference according to an unpaired Student *t*-test at a 99% confidence level).

The influences of the presence of glucose, the pH value, and the C/N ratio on the CCCP inhibitory effect were investigated. Indeed, carbon limitation, acidic pH, and nitrogen starvation are described as parameters that stimulate ligninases production [29]. As vanillin and vanillic acid appeared to have similar behaviors regarding their uptake, we focused these experiments on vanillin (Figure 2). By comparing the controls at pH 7 and C/N 21.34 with and without glucose, the preincubation of the mycelium without glucose over 16 h before the vanillin addition appeared to have decreased the vanillin uptake in a non-significant way. The relative incorporated vanillin quantity in the presence of CCCP did not appear to be affected by the absence of glucose in the considered experimental setup. The preincubation without glucose is susceptible to having significantly impaired intracellular ATP pools, which is likely to have direct consequences on the primary transport phenomena. In contrast, secondary active transports use gradients, which can persist for a limited time after ATP depletion. The absence of a synergic effect between the absence of glucose and CCCP on vanillin uptake inhibition rates (85% of inhibition with glucose and 75% without glucose) could thus be an argument in favor of the involvement of a secondary transport phenomenon.

The use of a more acidic medium in a pH range classically used for ligninases production [30] triggered a significant vanillin uptake decrease when comparing the controls with glucose. This result appears in contradiction with a symport mechanism for vanillin uptake with protons since the extracellular protons concentration was higher in the pH 5.5 medium, which reinforces PMF and would thus be expected to promote the vanillin uptake. However, the intracellular pH regulation may require more energy and act on vanillin uptake through an indirect process. The incorporated vanillin relative quantity with CCCP was not significantly affected by the decreased pH and appeared slightly higher than in other conditions with CCCP at C/N 21.34. By comparing it with the corresponding control at pH 5.5, it is interesting to note that the inhibition rate was around 50%, whereas it was 85% at pH 7 in the same glucose concentration and C/N ratio conditions. This change in the inhibition rate could be explained by a higher difficulty for CCCP to disrupt the PMF promoted by the higher extracellular proton concentration. This result would also be in agreement with the hypothesis of a secondary transport mechanism. Nevertheless, it can be noticed that relative incorporated vanillin quantities were relatively constant with CCCP between all the experiments and it could thus correspond to minimal retention by mycelium, for instance, due to biosorption, which would disturb the comparisons of differences with the controls. Thus, if this experimental approach enables clearly showing the involvement of active transport in vanillin uptake by *P. chrysosporium*, it is not sufficient to be conclusive regarding the precise mechanism. As the main objective of this study was to optimize the monophenols harvesting after fungal depolymerization, the main information given by this experiment was that an acidic pH that is suitable for ligninases production enables having incorporated vanillin relative quantities with CCCP as low as with pH 7. Within the same objective, a nitrogen limitation that has also been described as favoring ligninases production [31] was tested to see the consequences on the CCCP inhibitory effect (Figure 2). Similar to the pH decrease, the C/N ratio increase appeared to impair the vanillin uptake in the controls but had no significant effect on the vanillin incorporated quantities with CCCP. The CCCP still triggered a statistically significant inhibition around 60% when compared to the corresponding control at a C/N ratio of 213.4. Here again, the main objective was to decrease the monophenols uptake, while the main information of this result is that the inhibition protocol was compatible with a higher C/N ratio.

The main observations in relation to the aim of this study were that the uptake of lignin derivable monophenols models relied on an active transport phenomenon, which could be inhibited and that the CCCP was able to significantly decrease the monophenols uptake in an intact living mycelium. Indeed, the previous study of Shimizu et al. [19] had more fundamental goals and used fungal protoplasts, which are not compatible with our objective of the optimization of fungal lignin depolymerization. Furthermore, the developed inhibition protocol was found to be compatible with culture conditions that are more suited to ligninases production (acidic pH and higher C/N ratio) to evaluate its influence on lignin depolymerization.

### 3.3. Effect of Phenolics Uptake Inhibition on DHP Depolymerization

The influence of the protocol of phenolics uptake inhibition on the lignin depolymerization activity of the fungi was investigated on simplified models of lignin. This is an important aspect to study before further development of the protocol of monomers production since the lignin depolymerization by fungi occurs through extracellular enzymes, which could have their production or their excretion disturbed by the use of a metabolic inhibitor.

**Figure 3:**
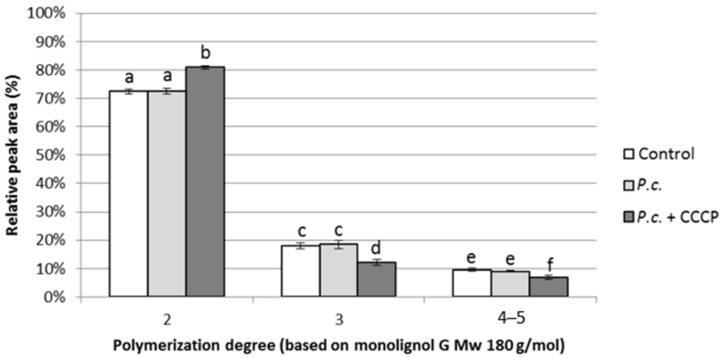
Influence of CCCP on the ability of *P. chrysosporium* (*P.c.*) to depolymerize dehydrogenation polymers (DHPs) at pH 5.5 and a C/N ratio of 21.34 (letters indicate groups with no significant statistical difference according to an unpaired Student *t*-test at a 99% confidence level).

A widespread model of lignin, known as DHPs, was synthesized and the impact of CCCP on its depolymerization was evaluated (Figure 3). The degradation of DHPs was followed by HPSEC. The mass distribution of DHPs in the controls was not modified in a significant way when the depolymerization was conducted with *P. chrysosporium* alone. When CCCP was added to the cultures with *P. chrysosporium*, the smallest oligomers were accumulated in a significant way compared to both the controls and the fungus alone, thus confirming the inhibition of internalization mechanism. Interestingly, the proportion of big oligomers significantly decreased compared to the controls and the fungus alone. This means that the developed protocol of the phenolics uptake inhibition through CCCP did not appear to impair the depolymerization capacities of the fungus but could even improve it. A plausible hypothesis is that the lack of monomers uptake could upregulate the production of extracellular ligninases in the cell to improve the lignin depolymerization efficiency and substrate supply. Interestingly, the enzymatic activity assays showed an increase of extracellular peroxidase activities when *P. chrysosporium* was incubated with CCCP and DHP in comparison with the culture of the fungus with DHP and without CCCP (Supplementary Materials). Modification of the C/N ratio to 213.4 triggered the same trends in a less significant way (Supplementary Materials) and was thus not used in the experiments with purified lignins.

### 3.4. Applicability of the Developed Inhibition Method on Purified Lignins

**Figure 4:**
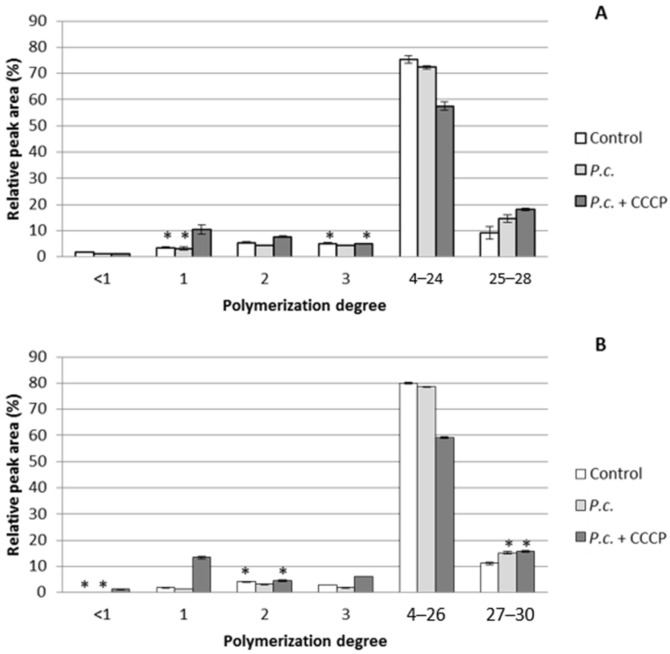
Impact of *P. chrysosporium* (*P.c.*) with and without CCCP on the polymerization degree of Kraft (**A**) and Organosolv (**B**) lignins after 11 days of incubation in a mineral medium at pH 5.5 and a C/N ratio of 21.34 (asterisks indicate averages that were not statistically different according to an unpaired Student *t*-test at a 99% confidence level for the same polymerization degree).

*P. chrysosporium* was incubated with and without CCCP with two purified industrial lignins (Kraft and Organosolv) for 11 days (Figure 4).

When incubations were conducted without CCCP, only small variations were observed compared to the controls with a decrease of both low molecular weight oligomers (1–3 monomers) and fractions 4–24/26. Thus, *P. chrysosporium* seemed to depolymerize fractions 4–24/26 and to metabolize the available small oligomers as their production progresses, which maintained the concentration of small oligomers at very low levels. CCCP favors the accumulation of monomers in a significant way and, to a lesser extent, of dimers and trimers for both Organosolv and Kraft lignins, confirming the efficiency of the developed protocol to inhibit oligomers uptake. In more detail, CCCP enabled the accumulation of around 3 times more monomers from Kraft lignin than in both the controls and the tests with the fungus. In the case of Organosolv lignin, the monomers accumulation due to CCCP reached 7 times the level of the controls and 10 times that of the tests with the fungus alone. Concerning higher polymerization degrees, the presence of the inhibitor appears not to have disturbed the depolymerization of fractions 4–26, but even stimulated it. This phenomenon could be explained by an upregulation of the ligninases production due to the inhibition of the small oligomers uptake, as was already hypothesized with DHP. The relative increase of the highest masses (fractions 27–30) with the fungus was likely due to the repolymerization phenomena. The depolymerization of fractions 4–26 and the accumulation of monomers were slightly higher for Organosolv lignin than Kraft lignin. The harsh treatment during the Kraft process is known to favor the formation of carbon–carbon bonds within lignin, leading to a recalcitrant polymer [8]. On the other hand, Organosolv lignin was reputed for the softness of the process, leading to the limited modification of natural lignin [32]. The acquisition of statistically significant results in such a short incubation time with both a purified lignin described as being close to the original lignin structure and a lignin reputed as more recalcitrant to the depolymerization is promising for the optimization of a depolymerization protocol for untreated lignocellulose on larger scales.

## 4. Conclusions

The use of the metabolic inhibitors sodium azide and CCCP enabled the confirmation of the involvement of an active transport mechanism in the lignin-derived monophenols uptake within *P. chrysosporium* cells, which is likely to correspond to a secondary active transport. CCCP enabled not only an efficient inhibition of oligomers uptake but also a stimulation of the depolymerization of DHP and purified lignins, leading to the extracellular accumulation of valuable oligomers. Further studies are required to validate the applicability of the technique to lignocellulosic biomasses at larger scales.

## Supplementary Materials

The following are available online at https://www.mdpi.com/2309-608X/6/4/362/s1, Analysis procedure of HPSEC chromatograms, Extracellular peroxidases activities after 3 days and Results of DHP depolymerization with a C/N ratio of 213.4.
