# Supplementary material for: Inhibition of Phenolics Uptake by Ligninolytic Fungal Cells and Its Potential as a Tool for the Production of Lignin-Derived Aromatic Building Blocks
Source: J Fungi (Basel). 2020 Dec 12;6(4):362. doi: 10.3390/jof6040362 (PMC7770579; doi:10.3390/jof6040362)
Supplement: Supplementary file 1 [file jof-06-00362-s001.pdf]

Supplementary data

Inhibition of phenolics uptake by ligninolytic fungal cells and its potential as tool for the production of lignin-derived aromatic building blocks

Mathilde Leriche-Grandchamp[1]; Amandine Flourat[1]; Hangchen Shen[1,2]; Flavien Picard[1,2]; Heloïse Giordana[1,2]; Florent Allais[1] ; Antoine Fayeulle[2]*

[1] URD Agro-Biotechnologies Industrielles (ABI), CEBB, AgroParisTech, 51110, Pomacle, France

[2] Université de technologie de Compiègne, ESCOM, TIMR (Integrated Transformations of Renewable Matter), Centre de recherche Royallieu - CS 60 319 - 60 203 Compiègne Cedex, France

* Corresponding author: antoine.fayeulle@utc.fr

SD1: Analysis procedure of HPSEC chromatograms

To determine the abundance of each category of oligomers relatively to the whole components, the chromatogram of the control was divided in portions. Each portion corresponds to a peak (see below). To determine the size of the oligomers into the peak, the mass of each peak was determined in regard to polystyrene calibration curve, and then the determined mass was divided by the mass of a G unit (180 g.mol^{-1}) to approximate the oligomer size. Finally, the proportion of each peak was set as the area of the peak divided by the area of the whole chromatogram.

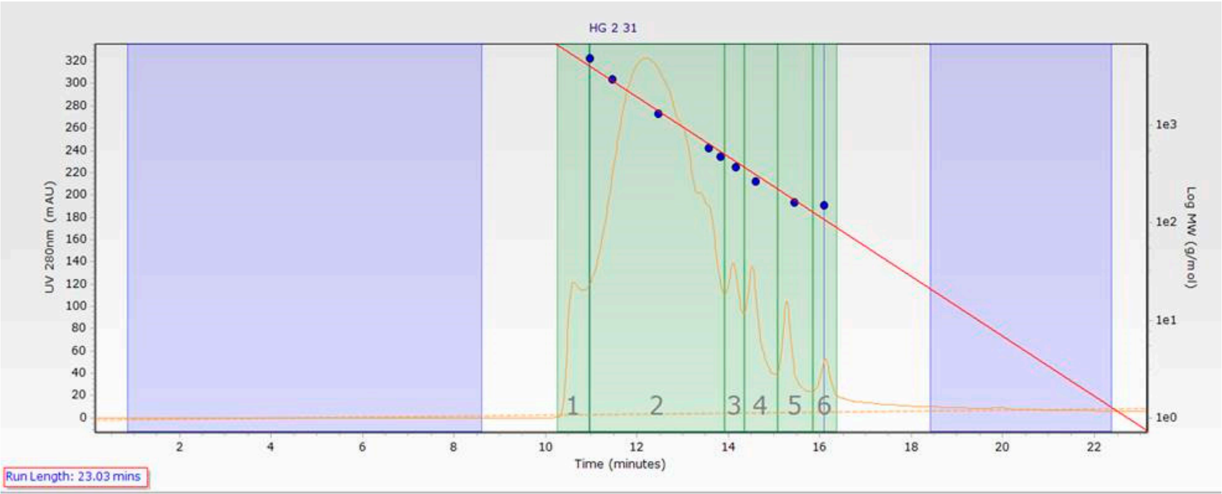

Figure SD1: HPSEC chromatogram of control assay on Kraft Lignin

Table SD1: Data from HPSEC chromatogram of control assay on Kraft Lignin

Peak	Average M_n	Average M_w	Peak area (%)	Oligomer size
1	4784	4837	6.37	25-28
2	1265	1653	76.85	4-24
3	432	435	5.42	3
4	301	307	5.89	2
5	179	182	3.72	1
6	106	107	1.74	>1

For the samples incubated with *Phanerochaete chrysosporium*, and those incubated with CCCP and *P. chrysosporium*, HPSEC chromatograms were divided with the same template as the control to track variation on the proportion of each class of oligomers.

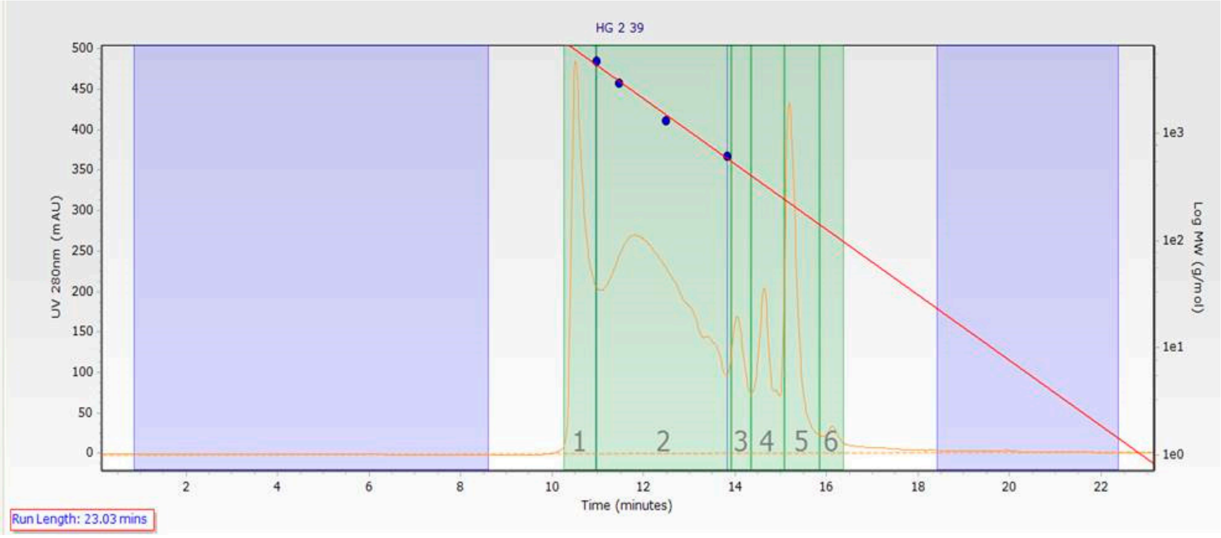

Figure SD2: HPSEC chromatogram of assay on Kraft Lignin incubated with CCCP and *Phanerochaete chrysosporium*

Table SD2: Data from HPSEC chromatogram of assay on Kraft Lignin incubated with CCCP and *Phanerochaete chrysosporium*

Peak	Average Mn	Average Mw	Peak area (%)	Oligomer size
1	5457	5532	17.82	25-28
2	1471	1992	56.09	4-24
3	479	482	5.16	3
4	300	307	8.04	2
5	209	211	11.76	1
6	118	119	1.14	>1

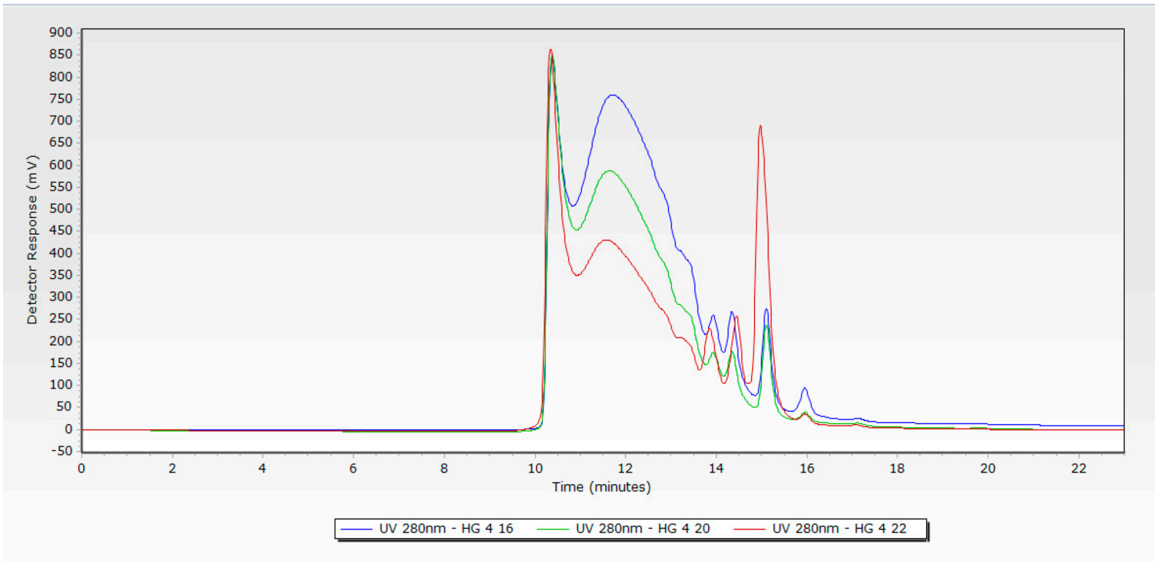

Figure SD3: Comparison between HPSEC chromatograms normalized on peak at 11 min from Kraft lignin for control (blue), incubation with *P. chrysosporium* (green) and incubation with *P. chrysosporium* and CCCP (red)

<u>**SD2:**</u> <u>Extracellular peroxidases activities after 3 days (measurement according to Zhou *et al.*, 2015)</u>

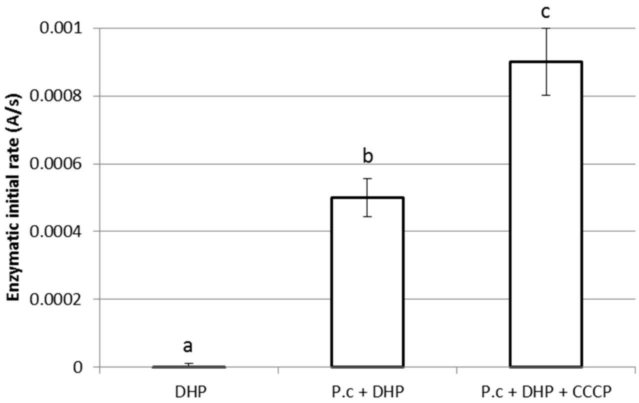

Figure SD4: Initial enzymatic rate of extracellular peroxidases after 3 days of incubation according to the conditions tested: DHP without the fungus, DHP + *P. chrysosporium*, DHP + *P. chrysosporium* + CCCP (average values of triplicates with error bars for the corresponding standard deviations; letters indicate groups with no significant statistical difference according to unpaired Student t-test at 99% of confidence).

SD3: Results of DHP depolymerization with a C/N ratio of 213.4

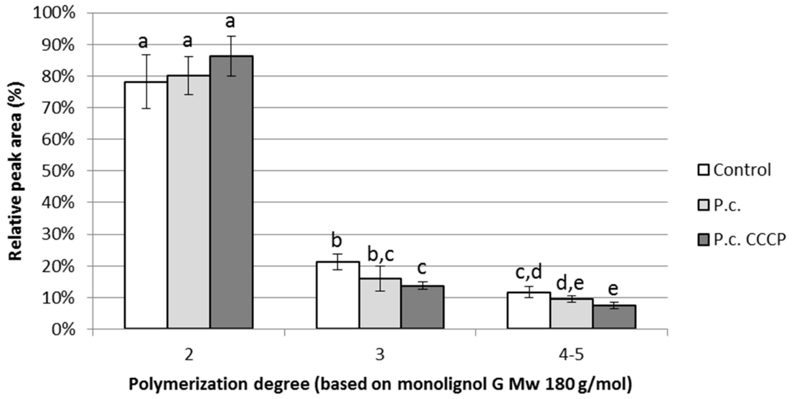

Figure SD5: Influence of CCCP on the ability of *P. chrysosporium* to depolymerize DHPs with pH5.5 and C/N ratio of 213.4 (letters indicate groups with no significant statistical difference according to unpaired Student t-test at 99% of confidence)
